# A case of compound X (black salve) self-treated melanoma in situ leading to hospitalization and extensive tissue damage

**DOI:** 10.1016/j.jdcr.2025.09.014

**Published:** 2025-09-25

**Authors:** Cindy Hakim, Camilla Cascardo, Elisabeth A. Pedersen

**Affiliations:** a Department of Dermatology, University of Michigan Medical School, Ann Arbor, Michigan; b Department of Dermatology, University of Michigan Medicine, Ann Arbor, Michigan

## Introduction

Compound X, also known as black salve, is an escharotic agent that is banned in several countries, including the United States. Despite regulation, it can be illegally or surreptitiously purchased, usually online, and is marketed as an alternative medicine for treating various benign and malignant skin conditions.^1^ Most formulations of black salve include the primary ingredients of bloodroot (*Sanguinaria canadensis*) and zinc chloride and induce tissue necrosis, leading to eschar formation and subsequent tissue sloughing.^2^ Despite increasing popularity among patients, black salve lacks clinical evidence supporting its efficacy and is associated with significant adverse events.^3^^,^^4^ These risks include severe tissue destruction, secondary infection, and delayed medical treatment. When black salve is used to treat malignancies, patients may experience incomplete tumor eradication, and this may also complicate treatment plans. In this report, we present a case of melanoma in situ (MIS) that a patient attempted to self-treat with black salve. Doing so resulted in cellulitis and full-thickness tissue necrosis requiring hospitalization and unnecessary tests, including a full-body positron emission tomography–computed tomography scan.

## Case report

**Fig 1:**
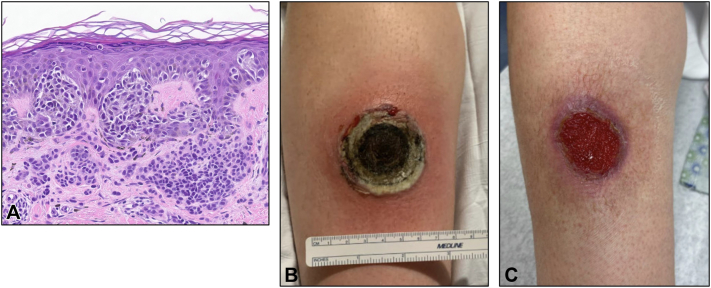
The patient had biopsy-proven melanoma in situ arising in association with a nevus on the right distal pretibial region **(A)**. A large, ulcerated lesion on the right distal pretibial region with overlying eschar surrounded by an erythematous rim after 23 days of treatment with black salve (compound X) **(B)**. Resolving ulcer on right distal pretibial region with central granulation tissue 1 month after hospitalization and intravenous antibiotics **(C)**.

A 38-year-old woman with a family history of multiple melanomas presented to the dermatologist with an 8-mm brown macule on the right distal pretibial region. A biopsy was performed and revealed atypical melanocytes arising within a background nevus, involving the peripheral margin (Fig 1, *A*). She was diagnosed with MIS, and a wide local excision was recommended. In the weeks preceding her scheduled excision, she attempted to treat the lesion at home with an herbal remedy known as compound X (black salve) in hopes she could avoid the procedure. The preparation was recommended by a family member who had reportedly used it to treat multiple melanomas in the past. She applied the salve to her skin daily for 23 days, noting erythema and “growth” of the lesion from day 1.

Two days after she stopped applying the compound, her lesion developed progressive pain, erythema, and ulceration within the treated area. She sought care in the emergency department and reported that she had been diagnosed with melanoma and was self-treating the area. Examination revealed a 5-cm wound with overlying eschar on the right shin, surrounded by erythema (Fig 1, *B*). The patient was admitted for intravenous antibiotics, and the inpatient team could not rule out lower extremity thromboembolism or melanoma progression, given the lack of specific information available about the patient’s melanoma history; therefore, computed tomography scans of the lower extremity, chest, abdomen, and pelvis were performed. This revealed chest nodules of unclear etiology. Dermatology was subsequently consulted due to concern for a rapidly progressing melanoma. The original melanoma pathology report was requested, but was unavailable until the next day. Imaging with total body positron emission tomography–computed tomography was performed in the interim, which did not reveal findings concerning malignancy. Upon obtaining the outside pathology report the next day (confirming MIS), and in light of the reassuring imaging studies, additional melanoma-related studies were deferred. She was treated for cellulitis and tissue necrosis and was discharged home with oral antibiotics and conservative wound care.

At follow-up 1 month later, the ulcer showed significant improvement with healthy granulation tissue formation and no clinical evidence of residual MIS (Fig 1, *C*). Her case was presented at our institution’s multidisciplinary cutaneous oncology tumor board. The National Comprehensive Cancer Network guidelines do not clearly outline a protocol for treatment of MIS with destructive injury, and the institutional consensus was that re-excision would likely cause additional morbidity without clear benefit given the degree of prior tissue destruction. Re-excision was not recommended unless the lesion recurred clinically, and the patient was advised to undergo close dermatologic surveillance.

## Discussion

Here, we present a case of self-treatment of MIS with compound X that not only highlights direct adverse effects of unregulated skin cancer treatment use and its unclear efficacy but also demonstrates indirect harms, including unnecessary testing and imaging leading to elevated costs, radiation exposure, and diagnostic confusion.

Historically, escharotic pastes with zinc chloride and botanicals were used for chemical cauterization in skin cancer treatment. Frederic Mohs first utilized a zinc chloride-based paste in fixed-tissue micrographic surgery before developing the fresh-tissue method that led to modern Mohs micrographic surgery.^1^^,^^2^ Despite historic use, regulatory agencies, including the US Food and Drug Administration and the Australian Therapeutic Goods Administration, have issued warnings against the use of black salve, citing potential adverse effects such as tissue necrosis, scarring, infection, and the risk of cancer progression due to inadequate treatment.^5^^,^^6^ The potential efficacy of black salve treatment was analyzed in an Australian study of 387 histopathology cases. Seventy-four basal cell carcinomas, 53 squamous cell carcinomas, 5 keratoacanthomas, 4 melanomas, and 1 case of cutaneous metastatic renal carcinoma were among the black salve-treated lesions. They reported that 35.2% of the specimens exhibited residual cancer following black salve treatment.^3^ The tumor type of the positive lesions was not reported. The authors further reported that between 2015 and 2019, the rate of black salve use had doubled. This may be in part due to social media, as Basch et al^7^ analyzed popular YouTube videos related to skin cancer and found that many consumer-generated videos promoted black salve as a home remedy, despite the lack of scientific evidence supporting its efficacy. These videos show before-and-after imagery and personal testimonials, misleading viewers about the safety and effectiveness of such treatments,^7^ and contribute to the concerning idea that black salve can substitute as “do-it-yourself Mohs”.^8^

While the use of black salve has not been rigorously studied, it sporadically appears in the medical literature in discussions of alternative therapies, and several case reports highlight the risks of use. One report described a patient who applied black salve to a superficial spreading melanoma with a Breslow depth of 0.6 mm for 24 hours, resulting in ulceration that the patient presumed to be complete treatment, ultimately leading to delayed care and progression to metastatic disease.^9^ Another case involved a woman who self-treated a presumed basal cell carcinoma on her nose with black salve, resulting in extensive tissue necrosis and complete loss of the left nasal ala.^10^

As black salve use continues to rise despite regulatory warnings and documented adverse effects, this case underscores the broader implications of online health misinformation related to dermatologic practice. Our case illustrates how black salve use resulted in thousands of dollars of additional health care expense to treat and work up the ulcer that it caused. This further reinforces the necessity of patient education, early intervention, and the promotion of scientifically validated treatment modalities to mitigate the risks posed by unproven alternative therapies.

## Conflicts of interest

None disclosed.
